# Freehand Minimally Invasive Pedicle Screw Fixation and Minimally Invasive Decompression for a Thoracic or Lumbar Vertebral Metastatic Tumor From Hepatocellular Carcinoma

**DOI:** 10.3389/fsurg.2021.723943

**Published:** 2021-12-01

**Authors:** Wenshuai Fan, Tianyao Zhou, Jinghuan Li, Yunfan Sun, Yutong Gu

**Affiliations:** ^1^Department of Orthopedic Surgery, Ruijin Hospital, Shanghai Jiao Tong University School of Medicine, Shanghai, China; ^2^Department of Orthopedic Surgery, Zhongshan Hospital, Fudan University, Shanghai, China; ^3^Department of Hepatic Oncology, Zhongshan Hospital, Fudan University, Shanghai, China; ^4^Department of Liver Surgery and Transplantation, Liver Cancer Institute, Zhongshan Hospital, Fudan University, Shanghai, China; ^5^Key Laboratory of Carcinogenesis and Cancer Invasion, Ministry of Education, Shanghai, China; ^6^Department of Orthopedic Surgery, Shanghai Public Health Clinical Center, Fudan University, Shanghai, China

**Keywords:** spinal metastasis, minimally invasive surgery, advantages, hepatocellular carcinoma, neurological decompression

## Abstract

**Objective:** To compare freehand minimally invasive pedicle screw fixation (freehand MIPS) combined with percutaneous vertebroplasty (PVP), minimally invasive decompression, and partial tumor resection with open surgery for treatment of thoracic or lumbar vertebral metastasis of hepatocellular carcinoma (HCC) with symptoms of neurologic compression, and evaluate its feasibility, efficacy, and safety.

**Methods:** Forty-seven patients with 1-level HCC metastatic thoracolumbar tumor and neurologic symptoms were included between February 2015 and April 2017. Among them, 21 patients underwent freehand MIPS combined with PVP, minimally invasive decompression, and partial tumor resection (group 1), while 26 patients were treated with open surgery (group 2). Duration of operation, blood loss, times of fluoroscopy, incision length, and stay in hospital were compared between the two groups. Pre- and postoperative visual analog scale (VAS) pain score, Oswestry Disability Index (ODI), American Spinal Injury Association (ASIA) grade, ambulatory status, and urinary continence were also recorded. The Cobb angle and central and anterior vertebral body height were measured on lateral radiographs before surgery and during follow-ups.

**Results:** Patients in group 1 showed significantly less blood loss (195.5 ± 169.1 ml vs. 873.1 ± 317.9 ml, *P* = 0.000), shorter incision length (3.4 ± 0.3 vs. 13.6 ± 1.8 cm, *P* = 0.000), shorter median stay in hospital (4–8/6 vs. 8–17/12 days, *P* = 0.000), more median times of fluoroscopy (5–11/6 vs. 4–7/5 times, *P* = 0.000), and longer duration of operation (204.8 ± 12.1 vs. 171.0 ± 12.0 min, *P* = 0.000) than group 2. Though VAS significantly decreased after surgery in both groups, VAS of group 1 was significantly lower than that of group 2 immediately after surgery and during follow-ups (*P* < 0.05). Similar results were found in ODI. No differences in the neurological improvement and spinal stability were observed between the two groups.

**Conclusion:** Freehand MIPS combined with PVP, minimally invasive decompression, and partial tumor resection is a safe, effective, and minimally invasive method for treating thoracolumbar metastatic tumors of HCC, with less blood loss, better pain relief, and shorter length of midline incision and stay in hospital.

## Introduction

Medical progress has increased the survival rate of cancer patients in recent years but has also led to an increase in metastatic lesions. Hepatocellular carcinoma (HCC) is common cancer worldwide with a great potential for metastatic spread (1). A total of 905,677 new cases of HCC and 830,180 death cases occurred worldwide, of which China alone accounted for about 50% (2). Bone metastases are the third most common site of metastases after liver and lung, and vertebrae are the most common site of bone metastasis (3, 4). Spinal metastasis mainly results in pain, pathological fracture, and paralysis due to neurologic compression, so more attention needs to be paid to its treatment.

Non-surgical treatment such as analgesia, corticosteroids, chemotherapy, or radiation therapy can relieve pain but cannot improve biomechanical stability (5). Percutaneous vertebroplasty (PVP) is a minimally invasive, radiologically guided therapeutic procedure that is performed to inject acrylic bone cement into the vertebral body. It can relieve pain (6) as well as strengthen the destroyed vertebra to prevent the vertebra from further collapse and neurologic compression (7). However, PVP alone is not optimal for spinal metastatic tumors accompanied with symptoms of neurologic compression, as neurological function cannot be improved by PVP without decompression (8). Therefore, neurological decompression and spinal tumor resection combined with internal fixation should be performed either from an anterior, posterior, or combined approach (9, 10).

In this study, we established a technique of freehand minimally invasive pedicle screw fixation (freehand MIPS, implanting pedicle screws through minimal access in a paraspinal sacrospinalis muscle-splitting (Wiltse) approach and fixing rods over pedicle screws through subcutaneous soft tissues and muscles) combined with PVP (11–13), minimally invasive neurologic decompression, and partial tumor resection through a mini-posterior midline approach (14, 15) to treat thoracolumbar metastasis from HCC with neurologic compression. This method was compared to open surgery through a posterior central approach to evaluate its feasibility, efficacy, and safety.

## Materials and Methods

### Patients

Patients with 1-level HCC metastatic thoracolumbar tumor and neurologic symptoms were retrospectively reviewed in our hospital between February 2015 and April 2017. Inclusion criteria: (1) definitive pathological or radiological diagnosis of HCC according to American Association for Study of Liver Disease guidelines, (2) 1-level thoracic or lumbar vertebral metastasis with neurologic compression on MRI and CT, which is consistent with neurologic symptoms and physical signs, such as pain, numbness, hypoesthesia, muscle weakness, or paralysis of lower limbs. Exclusion criteria: vertebral primary malignant tumors, osteoporotic fractures, or hemangiomas without nerve compression.

### Pre- and Post-procedural Imaging

All patients were evaluated by posteroanterior and lateral X-ray, CT, and MRI. CT and MRI were used to clarify: (1) the existence of cortical breakthrough and extra vertebral extension, (2) the type of lesion (osteoblastic, osteolytic, or mixed), and (3) the degree of vertebral collapse and neurological compression. T2-weighted MRI images were used to assess epidural spinal cord compression (ESCC): bone-only disease (grade 0), epidural impingement (grade 1a), thecal sac deformation (grade 1b), cord impingement (grade 1c), cord compression with cerebrospinal fluid visible (grade 2), and cord compression with no cerebrospinal fluid visible (grade 3) (16). CT images were used to evaluate the position of pedicle screws and cement leakage after surgery.

### Surgical Procedure

Patients were assigned to two groups: group 1, treated with freehand MIPS combined with PVP, minimally invasive neurologic decompression, and partial tumor resection; group 2, treated with open surgery through a posterior central approach.

After general anesthesia, patients were placed on two transverse pads in the prone position to achieve moderate overextension.

#### Group 1

**Figure 1:**
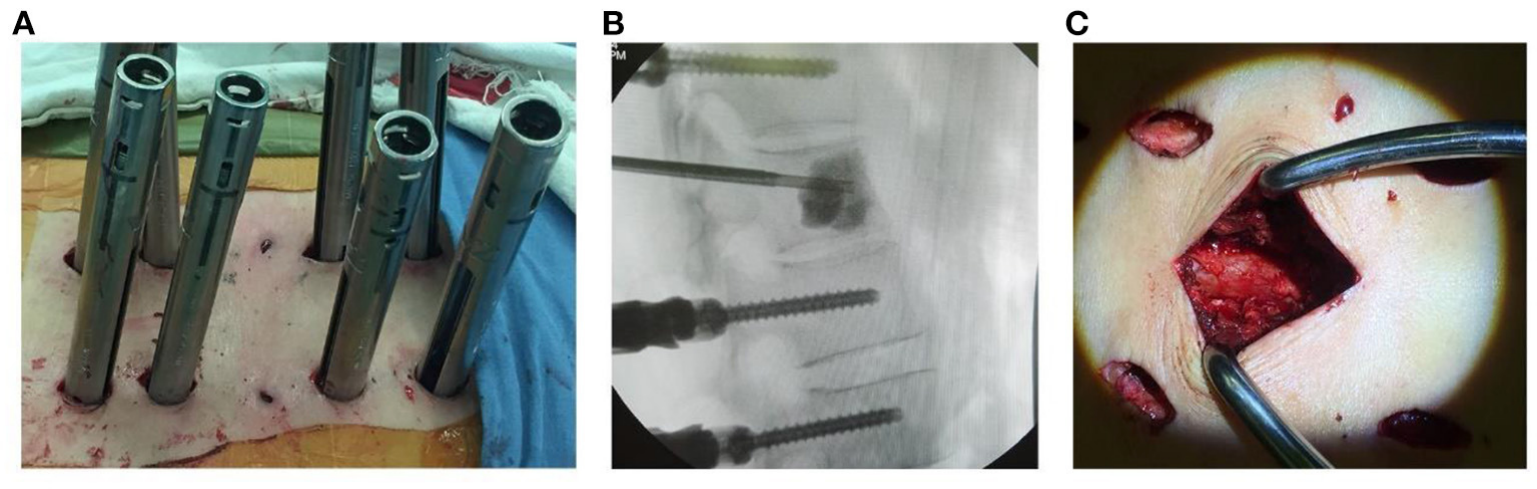
**(A)** Photography showing freehand minimally invasive pedicle screw fixation. **(B)** Injection of cement into the target vertebral body under fluoroscopic guidance. **(C)** Minimally invasive neurologic decompression and partial tumor resection.

Freehand MIPS+PVP (Figures 1A,B): After perspective positioning, incisions were made on the back 3.0 cm away from the midline (Wiltse approach). Each incision was 1.0 to 1.5 cm long. Above and below the involved vertebra, the adjacent 2-level vertebrae superior articular facet and root of transverse process were exposed through the multifidus interspace. The transverse processes of T11 and T12 were also exposed. Under direct vision, the entry site was located at the junction between the lateral border of superior articular facet and superior 1/3 line of the transverse process for thoracic pedicles, bisecting the midline of the transverse processes for T11 and T12 pedicles and the junction between the lateral border of superior articular facet and bisecting the midline of the transverse process for lumbar pedicles. Once the pedicle had been identified, a handheld curette was used to enter the pedicle and four pairs of pedicle screws (Viper, Depuy Spine, Raynham, Massachusetts, USA) of appropriate length were introduced into the vertebral body after the pedicle integrity was verified in four quadrants to ensure a solid tube of bone existed (11–13). Then two 13-gauge puncture needles were passed into the anterior central aspect of the involved vertebra through the pedicles under fluoroscopic guidance. Bone cement was injected into the target vertebra through puncture needles by a pressure injector (Shanghai Ruibang Biomaterials Co. LTD). Two rods were contoured according to the normal spine curve and placed over the pedicle screws through subcutaneous soft tissues.

Minimally invasive decompression and partial tumor resection (Figure 1C): A 3.5 cm midline incision was made to cut superficial and deep facial skin parallel to the involved vertebra with neurological compression. The paraspinal muscle was elevated subperiosteally, then the spinal process and lamina were removed to expose the dural sac involved in the neurological decompression. Facetectomies and pedicle resection were performed to obtain the posterior part of the vertebra with the rongeur or drill. Bipolar cautery and curettes or pituitary rongeurs were used for tumor piecemeal resection to create a cavity in the vertebra. The curved dura dissector was carefully inserted into the interface between the dura and tumor to push the posterior tumor compressing the neurological elements forward into the cavity of vertebra and create a “separation” of spinal cord and tumor for complete decompression, a procedure termed partial tumor resection (14, 15). Compression hemostasis was performed by packing absorbable hemostatic gauze into the cavity of vertebra. Attention should be paid to protect the spinal cord and nerve roots during the procedures.

#### Group 2

**Figure 2:**
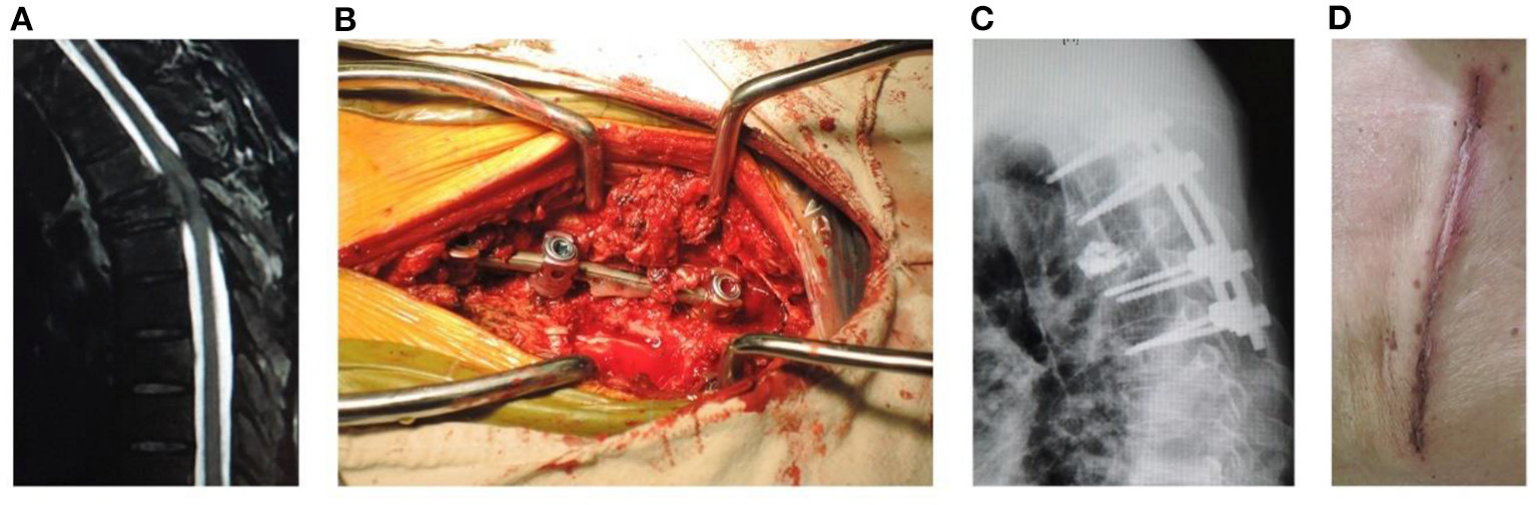
A male patient of 53 years old with T5 metastasis and neurologic compression from HCC had back pain VAS 8, ASIA C, Tomita 7, modified Tokuhashi 12, ESCC grade 3, and SINS 14. He underwent open surgery for internal fixation, PVP, neurologic decompression, and partial tumor resection, with an operation duration of 180 min, intraoperative blood loss of 800 ml, incision length of 14 cm, and stay in hospital of 11 days. **(A)** Sagittal MRI showed metastasis of T5 with neurologic compression. **(B)** Intraoperative photography. **(C)** Sagittal X-ray showed that the position of pedicle screws was good and no leakage of cement into the spinal canal occurred. **(D)** Incision after open surgery.

Open surgery for internal fixation, PVP, neurologic decompression, and partial tumor resection (Figure 2): A more than 10 cm midline incision was made to elevate the paraspinal muscle subperiosteally and expose the spinal process and lamina. The pedicle screws were inserted into the adjacent 2-level vertebrae above and below the involved vertebra. Bone cement was injected into the target vertebra through the puncture needles. Two contoured rods were placed over the pedicle screws. The spinal process and lamina were removed to expose the dural sac involved in neurological decompression; facetectomies and pedicle resection were performed to remove the posterior part of vertebra for partial tumor resection.

### Postoperative Management

If the physical condition of patients permitted, radiotherapy at the surgical site was performed 2 weeks after surgery when the incision healed to prevent local recurrence. Meanwhile, patients were recommended to the Department of Hepatic Oncology for comprehensive treatment, such as interventional therapy, chemotherapy, or targeted therapy.

### Clinical and Radiographic Evaluation

The Tomita Scoring System (17), Modified Tokuhashi scoring system (18), ESCC, and Spinal Instability Neoplastic Scoring (SINS) (16) were evaluated before the operation.

Back pain was measured with the visual analog scale pain score (VAS) before or immediately, 1, 2, 3, 6, and 12 months after surgery. The Oswestry Disability Index (ODI) was performed before or 3 and 12 months after surgery. American Spinal Injury Association (ASIA) classification of neurologic function, ambulatory ability, and urinary continence was also evaluated before and after surgery.

Anterior or central vertebral body heights and Cobb angles were measured on the lateral radiographs by the same doctor before or immediately, 1, 2, 3, 6, and 12 months after surgery. The spine was considered stable when no modification in the height of the vertebral body or Cobb angles was observed during the follow-up.

### Statistical Analysis

SPSS 25 software (SPSS Inc., Chicago, IL, USA) was used to perform statistical analysis. Age, interval from onset of neurologic deficit to surgery, duration of operation, times of fluoroscopy, cement injection, blood loss, incision length, stay in hospital, and follow-up period were compared between the two groups using independent-sample *t*-tests. Gender and metastasis location were compared using the chi-square test. Tomita scoring, Tokuhashi scoring, SINS, and ESCC were compared using the Mann-Whitney U test. VAS, ODI, Cobb angle, and the anterior or central vertebral body height were compared using a linear mixed-effects model for multiple comparisons. The 95% confidence level was used to define statistically significant differences.

## Results

Group 1 contained 21 patients who underwent freehand MIPS combined with PVP, minimally invasive decompression, and partial tumor resection. Group 2 included 26 patients treated with open surgery. Table 1 summarizes the comparison of clinical characteristics between the two groups. There were no significant differences in age (57.0 ± 10.4 vs. 60.2 ± 10.0, *P* = 0.290), gender (female/male, 8/13 vs. 9/17, *P* = 0.805), metastasis location (thoracic/lumbar, 14/7 vs. 16/10, *P* = 0.716), Tomita scoring (6–7/7 vs. 6–7/7, *P* = 0.823), Tokuhashi scoring (9–12/10 vs. 9–12/11, *P* = 0.875), SINS (6–14/11 vs. 6–14/12, *P* = 0.471), ESCC (2–3/2 vs. 2–3/2, *P* = 0.807), and interval from onset of neurologic deficit to surgery (1–7/3 vs. 1–7/3 days, *P* = 0.325) between the two groups. However, patients in group 1 showed significantly less blood loss (195.5 ± 169.1 vs. 873.1 ± 317.9 ml, *P* = 0.000), shorter incision length (3.4 ± 0.3 vs. 13.6 ± 1.8 cm, *P* = 0.000), and shorter stay in hospital (4–8/6 vs. 8–17/12 days, *P* = 0.000). Patients in group 1 showed significantly more times of fluoroscopy (5–11/6 vs. 4–7/5 times, *P* = 0.000) and longer duration of operation (204.8 ± 12.1 vs. 171.0 ± 12.0 min, *P* = 0.000). And there were no differences in the amount of cement injection (5.2 ± 0.8 vs. 5.0 ± 0.8 ml, *P* = 0.400).

**Table 1:** Comparison of clinical data between groups 1 and 2.

	**Group 1**	**Group 2**	***P*-value**
Age (years)	57.0 ± 10.4	60.2 ± 10.0	0.290
Gender (F/M)	8/13	9/17	0.805
Metastasis location (T/L)	14/7	16/10	0.716
Tomita (range/median)	6–7/7	6–7/7	0.823
Tokuhashi (range/median)	9–12/10	9–12/11	0.875
SINS (range/median)	6–14/11	6–14/12	0.471
ESCC (range/median)	2–3/2	2–3/2	0.807
Interval (range/median, days)	1–7/3	1–7/3	0.325
Duration of operation (min)	204.8 ± 12.1	171.0 ± 12.0	0.000
Fluoroscopy (range/median, times)	5–11/6	4–7/5	0.000
Cement injected (ml)	5.2 ± 0.8	5.0 ± 0.8	0.400
Blood loss (ml)	195.5 ± 169.1	873.1 ± 317.9	0.000
Incision length (cm)	3.4 ± 0.3	13.6 ± 1.8	0.000
Stay in hospital (range/median, days)	4–8/6	8–17/12	0.000
Follow-up period (months)	13.6 ± 2.2	11.7 ± 2.9	0.016

The back pain VAS significantly decreased after surgery in both groups (*P* < 0.05), especially in group 1. And VAS of group 1 was significantly lower than that of group 2 immediately and 1, 2, 3, 6, and 12 months after surgery (*P* < 0.05) (Table 2). The ODI also significantly decreased after surgery in both groups (*P* < 0.05). And ODI of group 1 was significantly lower than that of group 2 after surgery at 3 and 12 months (*P* < 0.05) (Table 3). Postoperative neurological functions improved in all patients. In group 1, 40% (2/5) of paralyzed patients experienced an increase of ASIA score from B to D, and 75% (12/16) of paraplegic patients experienced an increase of ASIA score from C or D to E at 3 month follow-up. A total of 13 (72%) of the 18 patients who survived 1 year after the surgery reached ASIA E. In group 2, 25% (2/8) of paralyzed patients showed an increase of ASIA from B to D, and 77% (14/18) of paraplegic patients showed an increase of ASIA from C or D to E at 3 month follow-up. Overall, 14 (70%) of the 20 patients who survived 1 year after the surgery reached ASIA E. In total, 4 (40%) of the 10 patients who could not walk before surgery regained the ability to walk after surgery in group 1, while 6 (42.9%) of the 14 patients in group 2 were able to walk again. The patients who could walk before surgery retained their ability. A total of 7 (53.8%) of the 13 patients with preoperative urethral sphincter dysfunction found that this function was restored in group 1, while 8 (50%) of the 16 patients regained this function in group 2.

**Table 2:** VAS pain assessment of the two groups.

**Group**	**Preoperative**	**Postoperative**	**1 month**	**2 month**	**3 month**	**6 month**	**12 month**
1	8.8 ± 0.9	3.0 ± 0.6	1.1 ± 0.8	0.7 ± 0.8	0.5 ± 0.5	0.5 ± 0.5	0.6 ± 0.5
2	8.4 ± 1.0	6.2 ± 0.8	3.2 ± 0.6	2.9 ± 0.6	3.1 ± 0.6	3.1 ± 0.6	3.1 ± 0.6
*P*-value	0.138	0.000	0.000	0.000	0.000	0.000	0.000

**Table 3:** ODI of thet two groups (%).

**Group**	**Preoperative**	**3 month**	**12 month**
1	84.3 ± 8.5	30.3 ± 9.4	21.1 ± 2.5
2	82.5 ± 9.1	38.2 ± 7.6	30.3 ± 3.5
*P*-value	0.485	0.003	0.000

**Figure 3:**
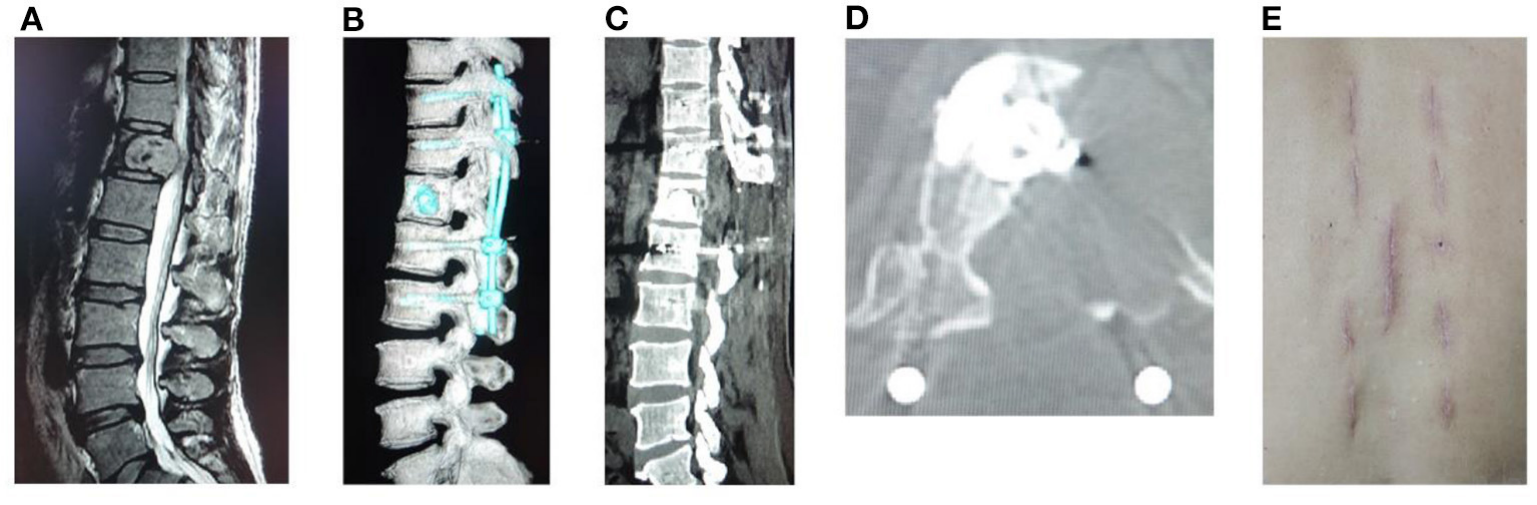
A male patient of 67 years old with T12 metastasis and neurologic compression from HCC had back pain VAS 9, ASIA B, Tomita 6, modified Tokuhashi 11, ESCC grade 3, and SINS 8. He underwent freehand MIPS+PVP, minimally invasive decompression, and partial tumor resection, with an operation duration of 215 min, intraoperative blood loss of 90 ml, incision length of 3.5 cm, and stay in hospital of 5 days. **(A)** Sagittal MRI showed metastasis of T12 with neurologic compression. **(B)** Sagittal CT reconstruction. **(C,D)** Sagittal and axial CT showed complete decompression and no leakage of cement into the spinal canal. **(E)** Incision after minimally invasive surgery.

Postoperative radiographs and CT scans demonstrated good positioning of pedicle screw constructs. No cement leakage occurred into the spinal canal. Decompression of the spinal canal was sufficient (a typical case in Figure 3). Spine stability was observed in all of the surviving patients at 1 year follow-up, and no hardware failure was seen in any patient. No significant differences were found in postoperative anterior or central vertebral body heights, and Cobb angles on spine X-rays during the follow-up between the two groups (Tables 4–6).

**Table 4:** Anterior vertebral body height of the two groups (mm).

**Group**	**Preoperative**	**Postoperative**	**1 month**	**2 month**	**3 month**	**6 month**	**12 month**
1	66.7 ± 7.5	67.2 ± 7.5	67.1 ± 7.5	67.1 ± 7.5	67.1 ± 7.5	67.1 ± 7.5	67.4 ± 7.5
2	66.2 ± 7.4	66.7 ± 7.5	66.7 ± 7.5	66.7 ± 7.5	66.7 ± 7.5	66.7 ± 7.4	66.8 ± 7.6
*P*-value	0.807	0.829	0.859	0.864	0.864	0.944	0.810

**Table 5:** Central vertebral body height of the two groups (mm).

**Group**	**Preoperative**	**Postoperative**	**1 month**	**2 month**	**3 month**	**6 month**	**12 month**
1	67.5 ± 6.7	68.1 ± 6.5	68.1 ± 6.5	68.1 ± 6.5	68.1 ± 6.5	68.1 ± 6.5	67.6 ± 6.8
2	66.0 ± 6.4	66.7 ± 6.3	66.7 ± 6.3	66.7 ± 6.3	66.7 ± 6.3	66.7 ± 6.5	67.2 ± 6.6
*P*-value	0.440	0.464	0.464	0.464	0.464	0.506	0.849

**Table 6:** Cobb angle of the two groups (°).

**Group**	**Preoperative**	**Postoperative**	**1 month**	**2 month**	**3 month**	**6 month**	**12 month**
1	9.1 ± 2.8	8.7 ± 2.8	8.7 ± 2.8	8.7 ± 2.8	8.7 ± 2.8	8.7 ± 2.8	9.1 ± 2.8
2	9.2 ± 2.9	8.9 ± 2.9	8.9 ± 2.9	8.9 ± 2.9	8.9 ± 2.9	8.9 ± 3.0	9.2 ± 2.9
*P*-value	0.912	0.877	0.877	0.877	0.877	0.876	0.928

## Discussion

Decision-making for surgical management of spinal metastasis is challenging because many factors need to be considered (19). Effective treatment to relieve cancer pain, save spinal cord function, and improve the poor quality of life is the clinical focus for patients with spinal metastasis. Minimally invasive spine surgery has become popular in recent decades with tremendous advancements in surgical techniques and technologies. It provides similar outcomes and shows lower perioperative adverse effects, and is considered a new treatment option over traditional open surgery (20, 21).

Paraspinal musculature iatrogenic injury is dramatically decreased during MIPS (11–14). Different from traditional midline incision, the Wiltse approach provides more direct access to transverse processes and pedicles, protecting the attachment of the muscle to bone, avoiding disruption of supraspinous and interspinous ligaments, and decreasing bleeding and postoperative pain. Compared to percutaneous pedicle screws, freehand MIPS uses incisions of similar size but with easier manipulation and less fluoroscopic projection during the operation. To further decrease aggressiveness and blood loss, a mini midline approach was used to perform minimally invasive neurological decompression and partial tumor resection (15).

Spinal surgery for metastatic tumors is associated with significant blood loss and the risk of catastrophic blood loss, especially for highly vascularized metastases, such as HCC (22). Though there is no consensus about the mean volume of blood lost during surgery for metastatic spinal disease, Chen et al. (23) conducted a meta-analysis and estimated that perioperative blood loss was 2,180 ml (95% confidence interval 1,805 to 2,554). Jung et al. (24) reported that the perioperative blood loss was 1534.4 ± 1484.2 ml in conventional open surgery (a posterior midline incision) for spinal metastases of HCC. Minimally invasive surgery has advantages in reducing blood loss in spinal surgery (25, 26). Our study showed that freehand MIPS, PVP, minimally invasive decompression, and partial tumor resection significantly decreased blood loss compared to the open operation. In addition, this surgery could be finished as soon as possible if there was massive blood loss during partial tumor resection and neurologic decompression.

Most patients with spinal metastasis presented severe pain, neurologic compression, and spinal instability, and the goal of surgery was not curing but relieving symptoms (27). Patients undergoing our minimally invasive treatment had better pain relief and ODI improvement than patients who underwent open surgery. The postoperative X-rays and CT scan images showed that complete neurologic decompression was achieved through the mini midline approach (Figure 3). Although it is difficult to reconstruct the anterior spinal column with a cage or other instruments, facetectomies and pedicle resection were performed to provide proper angulation to create a cavity in the vertebral body. Then the posterior tumor compressing neurologic elements was pushed forward into the cavity and separation of spinal cord and tumor was made for complete decompression (14, 15). Compared with open surgery, minimally invasive decompression and partial tumor resection through the mini midline approach can achieve comparable results in neurological improvement. Spine stability was also observed in all of the surviving patients at 1 year follow-up.

The postoperative X-rays and CT scan images confirmed the feasibility and safety of freehand MIPS combined with PVP, showing the proper position of screws and cement in patients of group 1. No postoperative neurologic complications were found in these patients. Ensuring a solid tube of bone exists during pedicle screw stabilization is crucial in avoiding the risks of nerve injuries (28). In our study, we implanted pedicle screws into vertebrae with the minimally invasive technique under direct vision to prevent violation into the spinal canal or the neuroforamen. Cement leakage into the spinal canal is another complication (29). During PVP, cement instillation was under constant fluoroscopy to avoid cement approaching the posterior aspect of the vertebral body or leaking into an extraosseous space. Though the fluoroscopic monitor was needed, the amount of radiation the patients received was as limited as that of PVP, because the freehand MIPS technique was performed under direct vision, without depending on fluoroscopy.

## Conclusion

Freehand MIPS combined with PVP, minimally invasive decompression, and partial tumor resection is a safe, effective, and minimally invasive method for treating thoracic or lumbar vertebral metastatic tumors of HCC with nerve compression. Our minimally invasive strategy not only showed less blood loss, better pain relief, and shorter length of midline incision and stay in hospital, but also had the same results in neurologic decompression and support for spinal stability compared to open surgery.

## Data Availability Statement

The raw data supporting the conclusions of this article will be made available by the authors, without undue reservation.

## Ethics Statement

The studies involving human participants were reviewed and approved by Ethics Committee of Zhongshan Hospital. Written informed consent for participation was not required for this study in accordance with the national legislation and the institutional requirements.

## Author Contributions

WF performed the statistical analyses and drafted the manuscript. TZ collected the data. JL revised the manuscript. YS and YG carried out the studies and revised the manuscript. All authors contributed to the article and approved the submitted version.

## Funding

This work was supported by the Science and Technology Commission of Shanghai Municipality (17411950302), the Shanghai Charity Cancer Research Center (2017-40), and the National Natural Science Foundation of China (81802320 and 82101654).

## Conflict of Interest

The authors declare that the research was conducted in the absence of any commercial or financial relationships that could be construed as a potential conflict of interest.

## Publisher's Note

All claims expressed in this article are solely those of the authors and do not necessarily represent those of their affiliated organizations, or those of the publisher, the editors and the reviewers. Any product that may be evaluated in this article, or claim that may be made by its manufacturer, is not guaranteed or endorsed by the publisher.
